# Separation of *Mycobacterium smegmatis* From a Mixed Culture Using the Cell Wall Binding Domain of D29 Mycobacteriophage Endolysin

**DOI:** 10.3389/fmicb.2020.01119

**Published:** 2020-06-05

**Authors:** Gokul Nair, Vikas Jain

**Affiliations:** Microbiology and Molecular Biology Laboratory, Department of Biological Sciences, Indian Institute of Science Education and Research, Bhopal, India

**Keywords:** mycobacteria, endolysin, mycobacteriophage, phage therapy, TB diagnostic

## Abstract

Pathological infection caused by *Mycobacterium tuberculosis* is still a major global health concern. Traditional diagnostic methods are time-consuming, less sensitive, and lack high specificity. Due to an increase in the pathogenic graph of mycobacterial infections especially in developing countries, there is an urgent requirement for a rapid, low cost, and highly sensitive diagnostic method. D29 mycobacteriophage, which is capable of infecting and killing *M. tuberculosis*, projects itself as a potential candidate for the development of novel diagnostic methods and phage therapy of mycobacterial infections. In our previous study, we showed that the cell wall binding domain [C-terminal domain (CTD)] located at the C-terminal end of the D29 mycobacteriophage LysA endolysin very selectively binds to the peptidoglycan (PG) of *Mycobacterium smegmatis* and *M. tuberculosis*. Here, by using *M. smegmatis* as model organism and by exploiting the PG binding ability of CTD, we have developed a method to isolate *M. smegmatis* cells from a mixed culture *via* magnetic separation. We show that green fluorescent protein (GFP)-tagged CTD (CTD-GFP) can bind to *M. smegmatis* cells *in vitro* after treatment with non-ionic detergent Triton X-100. Fluorescence-based assays show that CTD-GFP binding to *M. smegmatis* cells is highly specific and stable, and is not disrupted by an excess of either GFP or BSA. We further fused CTD with glutathione-S-transferase (GST) to generate CTD-GST protein and carried out an anti-GST antibody-mediated coating of CTD-GST on Dynabeads. This allowed us to perform successful magnetic separation of *M. smegmatis* from a mixed culture of bacteria having both Gram-negative and Gram-positive bacteria. Furthermore, the separated cells could be confirmed by a simple PCR. Thus our assay allows us to separate and identify *M. smegmatis* from a mixed culture.

## Introduction

Despite tremendous advancement in medical and clinical biology, mycobacterial infections remain one of the major global health concerns. *Mycobacterium tuberculosis*, the bacterium that causes tuberculosis (TB), is listed among one of the highest mortality-causing infection agents, and has caused nearly 1.5 million deaths in 2018 (Harding, 2020). Non-availability of efficient, rapid, and reliable detection methods for TB is one of the major contributors to this high death index (Caviedes et al., 2000). Most patients carrying mycobacterial infections who are diagnosed early can be cured due to recent advancements in the therapeutics (Sloan et al., 2013). Thus, the need of the hour is to have improved diagnostic methods for mycobacterial infections (McNerney et al., 2012). Currently, there are very few approved diagnostic tools available including molecular tests, smear microscopy, and culture-based tests (Liu et al., 2007; Lange and Mori, 2010). Many of these methods for mycobacterial detection fail to satisfy parameters such as accuracy, sensitivity, and reproducibility, and high throughput techniques require special expertise (De Cock et al., 2013).

Bacteriophage-based therapeutics and diagnostics have always been of interest due to their specificity toward target bacterium (Fischetti, 2008; Abedon et al., 2017). Phages are easy to culture, economic, and can be stored for long periods. Further, the host range for a given phage is narrow and is generally limited to either species or strain suggesting its highly specific nature. Due to these properties, bacteriophages are readily considered as potential tool for bacterial detection and identification (McNerney et al., 1998; Simboli et al., 2005; Schofield et al., 2012; Pohane et al., 2014).

Mycobacteriophage D29 is considered as one of the most potent mycobacteriophages with highly diverse mycobacterial host (Rybniker et al., 2006). To release phage progeny at the end of lytic cycle, the phage produces lytic proteins from its lytic cassette, which then mediate mycobacterial cell lysis by targeting cell envelope (Payne and Hatfull, 2012; Catalao and Pimentel, 2018). LysA of D29 phage lytic cassette specifically targets the peptidoglycan (PG) layer of mycobacterial cell envelope, and therefore plays a crucial role in host cell lysis (Payne and Hatfull, 2012; Pohane et al., 2014; Catalao and Pimentel, 2018). We have previously shown that D29 LysA is a multidomain structure having an N-terminal catalytic domain (NTD), lysozyme like domain (LD), and a C-terminal domain (CTD) that specifically binds to the mycobacterial cell wall PG (Pohane et al., 2014). Thus, CTD plays a crucial role in anchoring LysA to the PG layer thereby mediating its successful degradation and ultimately resulting in mycobacterial cell lysis.

Since PG is one of the major components of mycobacterial cell envelope, it acts as a novel target for the development of rapid and sensitive mycobacteria detection assay. We have previously shown that the CTD binds very specifically to both *Mycobacterium smegmatis* and *M. tuberculosis* PG, but does not bind to the PG of other bacteria (Pohane et al., 2014). Here we have developed a method wherein we use an engineered CTD protein to isolate and identify *M. smegmatis* from a mixed bacterial culture.

## Methods

### Bacterial Strains, Media, and Growth Conditions

*Escherichia coli* strain XL1-Blue (Stratagene) was used for all cloning experiments, and strain BL21(DE3) (Novagen) was used for protein expression and purification. *Mycobacterium smegmatis* mc^2^155 was grown in Middlebrook 7H9 medium (Difco) supplemented with 2% glucose, 0.05% Tween 80 at 37°C with constant shaking at 200 r/min. *E. coli* MG1665, XL1-Blue, BL21(DE3), and *Bacillus subtilis* cells were cultured in LB broth (Difco) at 37°C with constant shaking at 200 r/min. Wherever required, medium was supplemented with 100 μg/ml ampicillin. For solid medium culture, 1.5% agar was added to the growth medium while excluding Tween 80.

### Molecular Cloning

The plasmids used in this study are listed in Table 1. Oligonucleotides used in various PCR reactions are given in Table 2. Glutathione-S-transferase (GST) tag was added at the C-terminus of CTD by PCR amplifying CTD gene from pETGP10CTD using the oligonucleotides listed in Table 2, digesting the amplicon with *Eco*RI and *Bam*HI, and ligating it in pGEX-4T-2 vector at the same sites to yield pGEXCTD-GST.

**TABLE 1:** List of plasmids used in the present study.

Plasmid	Protein	Oligonucleotide	Template
^1^pETGP10CTD	CTD		
^1^pETGP10CTD-GFP	CTD-GFP		
pGEXCTD-GST	CTD-GST	CTD_GFP_GST_for	pETGP10CTD
		CTD_GST_Rev	

*The oligonucleotides and the template that were used to generate the respective clones are also mentioned. ^1^Source: Laboratory stock (Pohane et al., 2014).*

**TABLE 2:** List of oligonucleotides used in the present study.

Oligonucleotide	Sequence (5′–3′)	Purpose
CTD_GFP_GST_for	CCAGAGGATCCGGCGACCAACTACTGACTC	Cloning of
CTD_GST_rev	GGTGGAATTCTTGCGGCCGCTAGGGCTCCTGG	CTD-GST
mnoRTfor	TCTGCTTGTTGGTGGACTTG	Detection of *M. smegmatis*
mnoRTrev	GTCGAACCCCAAGGACTACA	by PCR
LacZ_gn_for	GCTGGAGTGACGGCAGTTATCTGGAAG	Detection of *E. coli*
LacZ_gn_rev	CAGAAACTGTTACCCGTAGGTAGTCACG	by PCR
rpoB_BS_gn_for	CCAAGGTACGTGCTACAACCAGCGTC	Detection of *B. subtilis*
rpoB_BS_gn_rev	CCTCATAGTTGTAGCCATCCCACGTC	by PCR

*Underlined sequences represent the restriction enzyme site.*

### Expression and Purification of Proteins

Plasmids pETGP10CTD and pETGP10CTD-GFP were used to express and purify CTD and CTD tagged with green fluorescent protein (CTD-GFP), respectively, as described previously (Pohane et al., 2014). For the purification of GST tagged-CTD (CTD-GST), *E. coli* BL21(DE3) carrying pGEXCTD-GST was cultured at 37°C with constant shaking at 200 r/min until the optical density of the culture at 600 nm (OD_600_) reached ∼0.6. The cells were then induced with 0.3 mM IPTG, and further incubated at 22°C with constant shaking at 150 r/min for 8 h. Cells were then harvested and resuspended in lysis buffer (40 mM Tris-Cl, pH 8.0, 400 mM NaCl, 5 mM 2-mercaptoethanol) and lyzed by sonication. The lysate was clarified by centrifugation at 18,000 r/min at 4°C for 1 h, and the supernatant was incubated with pre-equilibrated Glutathione Sepharose 4 Fast Flow matrix for 2 h. The matrix was washed with buffer containing 40 mM Tris-Cl, pH 8.0, 500 mM NaCl, 100 μM reduced L-glutathione, and 5 mM 2-mercaptoethanol. The protein was then eluted using a buffer containing 40 mM Tris-Cl pH 8.0, 200 mM NaCl, 10 mM reduced L-glutathione, and 5 mM 2-mercaptoethanol. The elution having ample amount of protein was subjected to dialysis in a buffer having 40 mM Tris-Cl pH 8.0, 200 mM NaCl, 1 mM dithiothreitol, and 40% glycerol. The protein was collected, centrifuged at 14,000 r/min for 15 min at 4°C, and analyzed on SDS-PAGE. The supernatant was stored at −20°C until further use.

### Mixed Bacterial Sample Preparation and Protein Binding Assays

Different test samples for assay were prepared to have different combinations of *M. smegmatis* mc^2^155, *E. coli* MG1665, and *B. subtilis* cells. All the bacteria were individually grown till OD_600_ reached ∼0.6, and normalized according to the requirement of the number of cells with respect to experimental setup. The samples were harvested by centrifugation at 8000 r/min at room temperature. The cell pellet was resuspended in 1 ml of binding buffer (25 mM Tris-Cl, pH 8.0, 200 mM NaCl) containing 1% Triton X-100 and incubated at 37°C with constant shaking at 800 r/min using a thermomixer (Eppendorf) for 2 h. Cells were again harvested by centrifugation and resuspended in binding buffer having 0.05% Triton X-100. Resulting cell suspension was then used in the protein binding assays by incubating various bacterial cells with 5 μg of various proteins (CTD, CTD-GFP, GFP, BSA). The proteins were allowed to bind to the cells by constant mixing at 800 r/min at 37°C for 2 h. Cells were harvested post-incubation by centrifugation and washed twice with binding buffer. For fluorescence microscopy, cells were serially diluted, added to an agarose pad (prepared by dissolving 0.5% agarose in TAE buffer and spreading it over a glass slide in the form of pad), and imaged on a fluorescence microscope (Leica DM 2500) under 100X objective with GFP filter. Alternatively, the cell pellet was either resuspended in binding buffer devoid of Triton X-100 for fluorescence measurements or resuspended in binding buffer with 0.05% Triton X-100 to carry out competition assays. 200 μl of the suspension devoid of Triton X-100 was subjected to fluorescence measurement on Spectramax M5 (Molecular Devices). For performing competition experiments, cells were incubated with 20 μg of the competitor protein with constant mixing at 800 r/min at 37°C for 2 h. Cells were again harvested post-incubation by centrifugation and washed twice with binding buffer devoid of Triton X-100, and were used for fluorescence measurements. Additionally, the cell pellet present in the tube was imaged by using a digital camera (Canon) and an amber filter while the tube was illuminated using a blue light transilluminator (∼470 nm).

### Magnetic Separation of *M. smegmatis* Cell

Purified CTD-GST protein was used for the *M. smegmatis* cell separation. Dynabeads M-270 Epoxy beads (10 mg) were coupled with the anti-GST monoclonal antibody (Thermo Fisher Scientific) using antibody coupling kit (Thermo Fisher Scientific) following manufacturer’s instructions to obtain a final bead concentration of 10 mg/ml. Anti-GST antibody-coupled Dynabeads were next equilibrated in binding buffer before protein coating. Equilibrated beads were incubated with 200 μg CTD-GST protein at 4°C overnight with gentle rotation thus allowing the protein to have efficient interaction with the antibody. The CTD-GST bound Dynabeads were equilibrated in binding buffer and eluted with final volume of 0.5 ml. The bead-protein complex (20 μl) was mixed with bacterial suspension containing *M. smegmatis* in combination with *E. coli and B. subtilis* as prepared above, and the mixture was incubated at 37°C with constant shaking at 800 r/min for 2 h. Next, the mixture was subjected to magnetic separation on a magnet bar (Thermo Fisher Scientific) by following manufacturer’s instructions, and the supernatant was collected. The magnetically separated sample was either directly used in PCR or was subjected to competition with CTD-GST protein in order to dissociate the *M. smegmatis* cells bound to Dynabeads, and the eluted cells were then subjected to PCR confirmation. PCR was carried out using Phusion enzyme (Thermo Fisher Scientific) and the oligonucleotides specific for the bacterium (Table 2). The amplicons were separated on agarose gel and imaged.

## Results

We have previously shown that CTD protein has specific PG binding property for mycobacterial PG (Pohane et al., 2014), which is one of the major components of the mycobacterial cell envelope. Here, we have explored the possibility of CTD to selectively bind to *M. smegmatis* cells directly in order to use this assay as a separation method for *M. smegmatis* cells from a mixed culture.

### C-Terminal Domain of LysA Binds to *M. smegmatis* Cells

**FIGURE 1:**
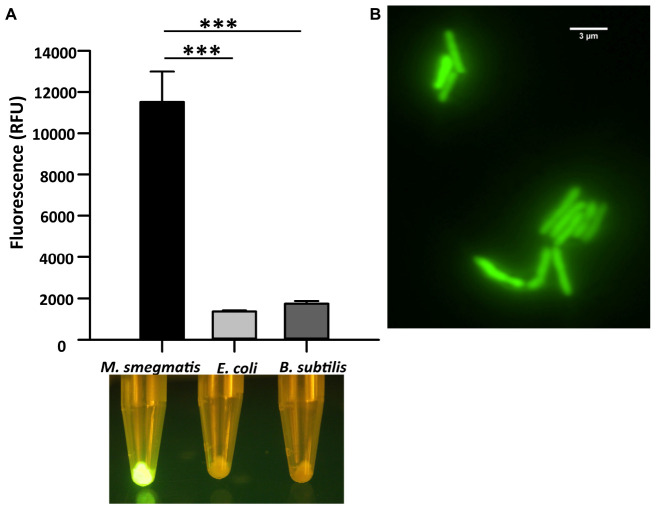
Cell binding assay of CTD-GFP with different bacterial cells. **(A)** The relative fluorescence obtained for CTD-GFP bound to *M. smegmatis*, *E. coli*, and *B. subtilis* cells. Fluorescence was measured by keeping the excitation and emission wavelengths at 488 and 509 nm, respectively. The data represent an average of three experiments with error bars denoting the standard deviation (*p*-value analysis: ^∗∗∗^, < 0.0003). The bottom panel shows the image of fluorescing cell pellet obtained after illuminating it with a blue light (∼470 nm) source. **(B)** Fluorescence microscopy imaging of CTD-GFP bound *M. smegmatis* cells. The image was taken on a Leica Microsystems fluorescence microscope with a GFP filter.

We first analyzed the *in vitro* binding of CTD of LysA specifically to *M. smegmatis* cells by constructing a GFP-tagged CTD protein. Here, GFP and hexa-histidine tag were added in tandem at the C-terminal of CTD. The recombinant protein was purified (Supplementary Figure S1), and was used for the *in vitro* binding assays. The purified protein was incubated with *M. smegmatis*, *E. coli*, and *B. subtilis*. The fluorescence of the GFP tag associated with CTD was visualized using a blue light transilluminator and also quantified on Spectramax M5 (Molecular Devices). Our data show that maximum fluorescence of CTD-GFP is obtained in the case of *M. smegmatis* only, whereas negligible fluorescence is observed from the other two bacteria (Figure 1A). The bacterial samples and CTD-GFP ratio in each case was kept constant to avoid any bias. We further examined CTD-GFP binding to *M. smegmatis* under the fluorescence microscope. The microscopic images demonstrate clear localization of GFP throughout *M. smegmatis* envelope (Figure 1B). Taken together, our data suggest that CTD-GFP is able to interact with intact *M. smegmatis* cells.

### CTD–*M. smegmatis* Cell Interaction Is Highly Specific and Stable

**FIGURE 2:**
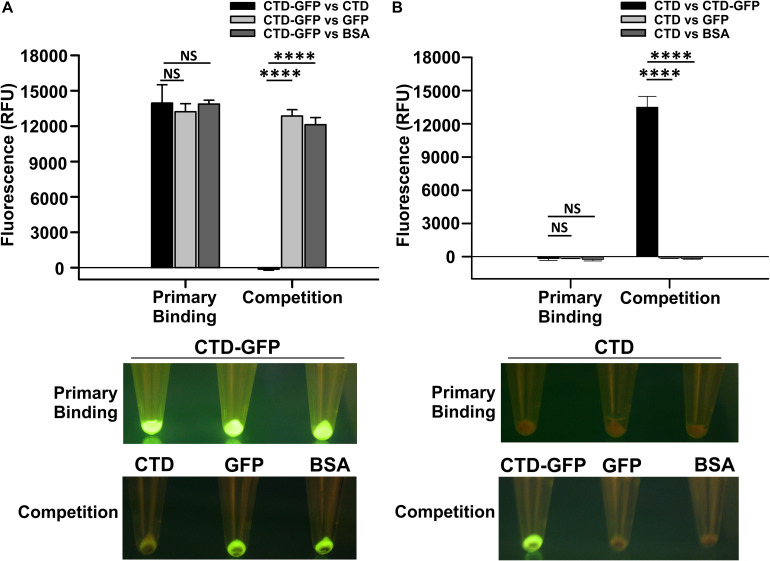
Qualitative and quantitative assessment of CTD binding to *M. smegmatis* cells. **(A)** The relative fluorescence obtained for *M. smegmatis* cells bound to CTD-GFP before (Primary binding) and after competitor addition (Competition). Competitors used are CTD, GFP, and BSA. **(B)** The relative fluorescence obtained for *M. smegmatis* cells bound to CTD before (Primary binding) and after competitor addition (Competition). Competitors used are CTD-GFP, GFP, and BSA. In both **A** and **B**, the data represent an average of three independent experiments with error bars denoting the standard deviation (*p*-value analysis: ^∗∗∗^, < 0.0001; NS, not significant). In both **A** and **B**, the bottom panels show the image of fluorescing cell pellet obtained after illuminating it with a blue light (∼470 nm) source before and after the competition.

Since CTD-GFP readily interacts with *M. smegmatis* cells, we asked if this interaction is both specific and stable. We, therefore, challenged the CTD-GFP–*M. smegmatis* cell interaction with non-specific competitors GFP and BSA as well as specific competitor CTD protein devoid of GFP (Supplementary Figure S1). First, cell binding assay was performed with *M. smegmatis* cells as a substrate and CTD-GFP protein as binding partner. Fluorescence of CTD-GFP-bound *M. smegmatis* cells were measured after multiple washes with binding buffer (Figure 2A). Next, the CTD-GFP protein bound to *M. smegmatis* cells was challenged with higher amounts of competitors, *viz*., CTD, GFP, and BSA proteins. Relative fluorescence measurements clearly show a significant decrease in fluorescence when the binding reaction was challenged with CTD, which is in sharp contrast with other two competitor proteins (Figure 2A). This competition was also clearly visible when the cells were illuminated on a blue light transilluminator to observe GFP fluorescence (Figure 2A). Only the cell pellet where the CTD-GFP was competed out with CTD did not show any fluorescence suggesting the complete displacement of CTD-GFP with CTD.

The above experiment was also attempted in reverse order also to rule out the possibility of GFP binding non-specifically to *M. smegmatis* cells. Here, we first incubated *M. smegmatis* cells with GFP-less CTD, and then challenged the interaction with CTD-GFP, GFP, or BSA. Our data show that only CTD-GFP was able to compete out CTD bound to *M. smegmatis* cells, thus resulting in a significant increase in relative fluorescence; GFP was unable to show this phenomenon and therefore, no fluorescence was observed in this case when subjected to blue light illumination (Figure 2B). This observation was also corroborated by the imaging of cell pellets treated with CTD and competitors; here, only the cell pellet with CTD-GFP as competitor showed fluorescence. These data additionally suggest that GFP does not interact with *M. smegmatis* cells. Taken together, our data show that CTD has a high affinity toward, and shows stable *in vitro* interaction with, *M. smegmatis* cells.

### Designing and Validation of CTD-Based *M. smegmatis* Detection Assay

**FIGURE 3:**
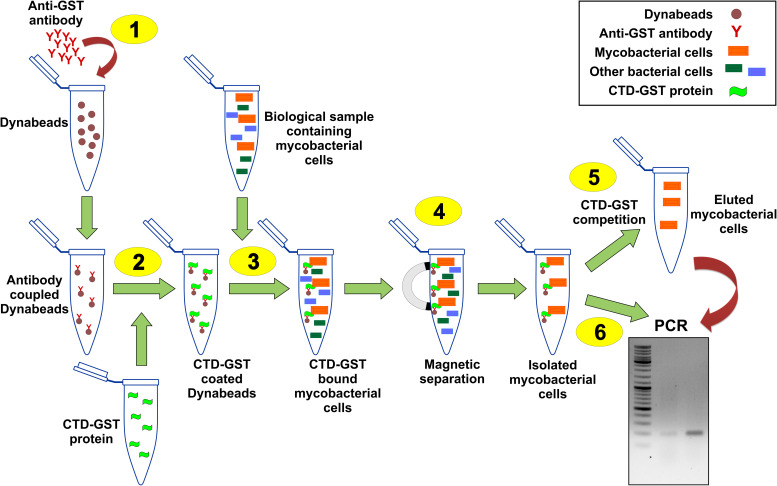
Schematic of CTD-based mycobacterial detection assay. Illustration here shows the general procedure for the isolation of *M. smegmatis* cells present in a given biological sample. 1–6 represent the key steps in the assay as detailed in “Methods” section. Step 1 is the coupling of anti-GST antibodies with Dynabeads. CTD-GST protein is then coated on these beads (Step 2). Step 3 involves the incubation of mixed bacterial sample having *M. smegmatis* cells with the product of step 2. Step 4 involves magnetic separation of the Dynabeads that pulls down *M. smegmatis* cells. The isolated cells can either be eluted by competition with CTD-GST (Step 5) or be used directly in a PCR (Step 6).

Figure 3 shows the schematic of the *M. smegmatis* cell separation and detection. To develop this assay, we generated CTD-GST protein construct, and used the purified protein (Supplementary Figure S1) in our experiments. Because of their magnetic property, Dynabeads were used in this method. These beads were coupled with anti-GST monoclonal antibody by chemical conjugation. The antibody-coupled Dynabeads were first incubated with CTD-GST protein, and, then the protein coated beads were mixed with a bacterial suspension containing *M. smegmatis* cells. The bacterial cells could then be readily separated by using a magnet bar. Here, centrifugation is avoided as that will result in the sedimentation of all the cells, whereas magnetic separation allows the separation of only CTD-GST-bound cells. *M. smegmatis* cells thus isolated were confirmed by PCR. As a proof of concept, by using a bacterial suspension containing other bacteria besides *M. smegmatis*, we demonstrate that the method developed here is applicable to mixed culture as well.

**FIGURE 4:**
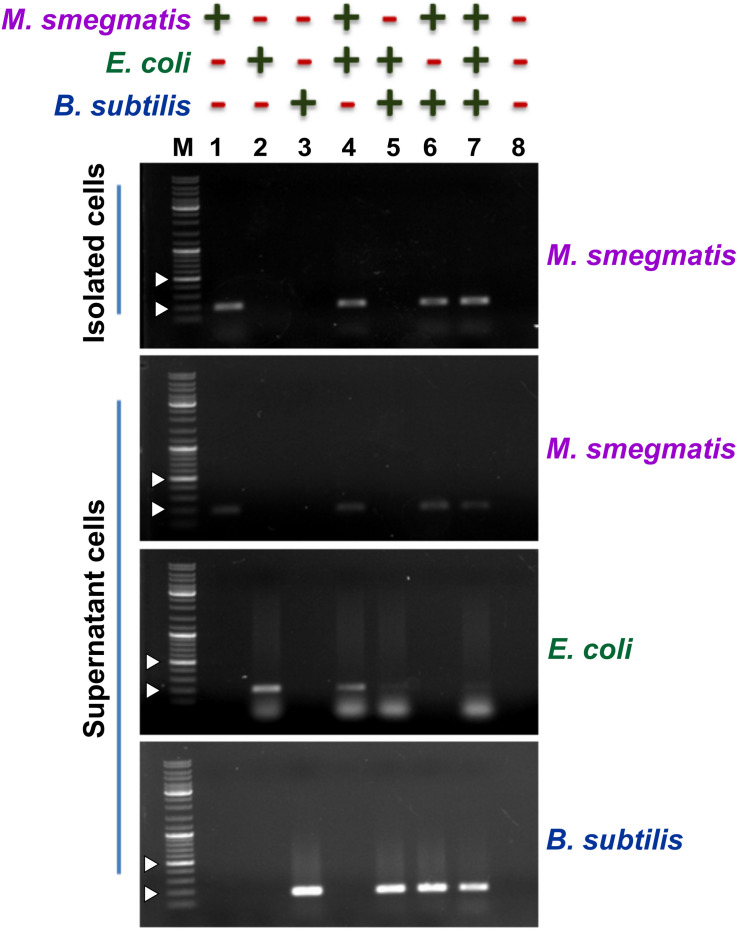
Detection of the presence of bacteria after magnetic separation from mix-samples. The assay was carried out with a bacterial sample having presence (+) or absence (−) of *M. smegmatis*, *E. coli*, and *B. subtilis* as indicated. Agarose gels show the PCR amplicon obtained after carrying out PCR with either the pulled-down cells or the cells present in the supernatant after magnetic separation. Lanes 1–8 represent all the different combinations of samples. PCR was performed using *mno* gene primer for *M. smeg*matis, *lacZ* gene primer for *E. coli*, and *rpoB* gene primer for *B. subtilis*. Obtained amplicon sizes are 188 bp for *mno*, 210 bp for *lacZ*, and 161 bp for *rpoB*. “M” represents the DNA ladder with two bands of 200 and 500 bp marked with white arrowheads.

Next, to assess the ability of the designed assay to selectively capture *M. smegmatis* cells, we used *M. smegmatis* cell suspension containing other Gram-positive and Gram-negative bacteria (Figure 4). In all, we prepared eight different cell suspensions having one or more of the following three bacteria—*M. smegmatis*, *E. coli*, and *B. subtilis*. Here, ∼10^4^ CFU/ml *M. smegmatis* cells were mixed with an equal proportion of *B. subtilis* and/or *E. coli* cells resulting in an equal final ratio of all bacterial cell types in the sample. All the samples were processed through the designed assay (Figure 3). Additionally, all the conditions were kept the same for all the samples to avoid any biasness. The eluted fraction after magnetic separation and the supernatant fraction from all the samples were subjected to PCR using bacteria-specific oligonucleotides. Our data show that out of the eight different combinations of bacterial samples, an amplicon with *M. smegmatis* specific oligonucleotides is observed only in the samples containing *M. smegmatis* cells (Figure 4). This immediately suggests a successful capture of *M. smegmatis* cells from a mixture having different combination of *M. smegmatis*, *E. coli*, and *B. subtilis*. Moreover, in the separated cells, the other bacteria could not be detected, which immediately suggests that the developed method is very specific. The supernatant obtained after magnetic separation was also harvested and subjected to PCR confirmation with primers specific for *M. smegmatis*, *E. coli*, and *B. subtilis*. In all the cases, the presence of the PCR amplicon on agarose gel clearly indicates and validates the presence of respective bacterium in the sample. Thus, the PCR confirmation of the presence of all bacteria before magnetic separation and the detection of only *M. smegmatis* cells post-magnetic separation clearly demonstrate a successful separation of only *M. smegmatis* cells from the samples having a mixture of bacteria.

### Evaluating the Capability of Designed Detection Assay

**FIGURE 5:**
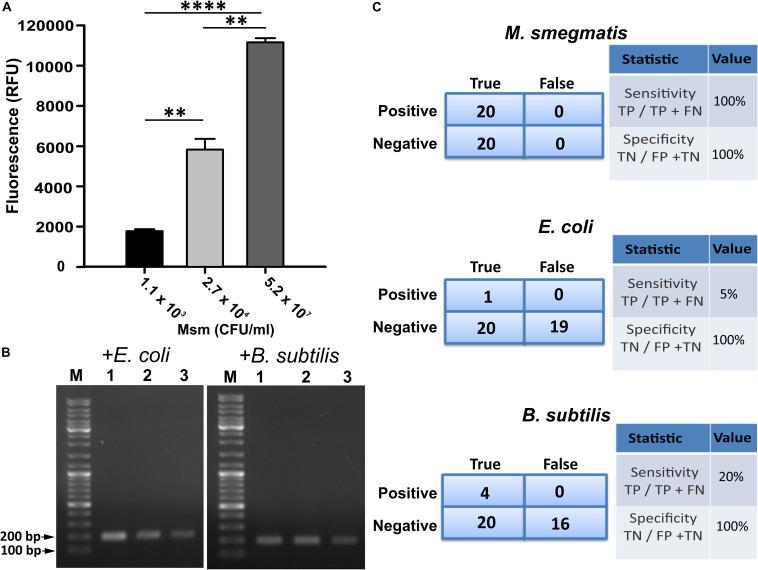
Evaluation of efficiency of designed *M. smegmatis* detection assay. **(A)** CTD-GFP was used for differential quantitative assessment of the presence of *M. smegmatis* in bacterial samples carrying a mixture of varying amount of *M. smegmatis* cells spiked with *E. coli* and *B. subtilis*. *x*-axis represents the CFU/ml of *M. smegmatis* (Msm) present in the sample for assay and *y*-axis represents fluorescence obtained due to CTD-GFP binding to *M. smegmatis* cells (*P*-value analysis: ^∗∗∗^, < 0.0001; ^∗∗^, < 0.0002). **(B)** Limit of detection of designed assay analyzed with minimal level of *M. smegmatis* spiked with either *E. coli* or *B. subtilis* as depicted above agarose gel images. Shown are the amplicons obtained by PCR carried out using *mno* gene primer. Lanes 1, 2, and 3 represent 1.1 × 10^3^, 2.7 × 10^4^, and 5.2 × 10^7^ CFU/ml of *M. smegmatis* cells, respectively, present in the samples. “M” represents the DNA ladder with two bands marked. **(C)** Statistical analysis of the specificity and the sensitivity of the detection of *M. smegmatis*, *E. coli*, and *B. subtilis*, as depicted. Experiments were performed multiple times to obtain statistically significant data. “TP,” “TN,” “FP,” and “FN” represent true positive, true negative, false positive, and false negative, respectively.

Since GFP fluorescence can be easily quantified, we used CTD-GFP protein to assess the presence of varied amount of *M. smegmatis* cells in different samples. We incubated different amount of *M. smegmatis* cells with CTD-GFP, and assessed protein binding by measuring the fluorescence after extensive washing of the cell pellet. Our data show a significant fluorescence with samples having 1.1 × 10^3^ CFU/ml *M. smegmatis* cells (Figure 5A); with an increase in number of cells, a significant increase in fluorescence is also observed (Figure 5A), which indicates that CTD-GFP can be useful as a potential reporter for the varying levels of *M. smegmatis* cells. We next estimated the efficiency of our assay using the limit of detection as one of the parameters by analyzing the minimum number of cells that can be detected. Samples carrying different amounts of *M. smegmatis* cells mixed with either *E. coli* or *B. subtilis* were subjected to our designed assay, and the presence of *M. smegmatis* cells was confirmed by PCR. Our data show that *M. smegmatis* could be detected in the bacterial samples carrying as low as 1.1 × 10^3^ CFU/ml (Figure 5B). We thus conclude that our designed assay has a limit of detection in range of 10^3^ *M. smegmatis* cells. We wish to add here that previous studies of mycobacterial detection assay suggest a detection limit of *M. tuberculosis* in the range of 10^5^ CFU/ml to be considered as significant (Park et al., 2009). Thus, our detection limit of 10^3^ CFU/ml in a mixed bacterial culture can be considered as very significant. Figure 5C presents the statistical analysis of the sensitivity and specificity of our assay in laboratory conditions. We find that out of the 20 samples analyzed, our assay shows 100% sensitivity and specificity toward *M. smegmatis* in comparison with *E. coli* and *B. subtilis*.

## Discussion

Rapid diagnosis of mycobacterial infections such as TB will significantly reduce the disease burden. Several classical and contemporary approaches are being used clinically to detect mycobacterial infections such as smear microscopy, culture identification, histopathology, tuberculin skin test (TST), serological assays, interferon-gamma release assays (IGRAs), and nucleic acid amplification (NAA) tests (Liu et al., 2007; Lange and Mori, 2010). Smear microscopy, although is one of the commonly used methods, has drawbacks owing to low and variable sensitivity. Similarly, culture identification is time-consuming with a turn-around time of 2–10 weeks (Padmavathy et al., 2003; Haldar et al., 2011; Derese et al., 2012). On the other hand, tissue samples analysis for mycobacterial infection using histopathological approach depends on the presence of granulomatous inflammation and caseous necrosis, and is therefore difficult and suffers from handling error with significant variability (Bravo and Gotuzzo, 2007; Haldar et al., 2011).

PCR-based approaches are considered to be one of the most accurate ways to detect mycobacteria. Not only does it shorten the turn-around time, automation of the procedure also reduces handling error and allows for the differentiation between different types of mycobacterial infections due to availability of novel marker genes for different mycobacterial strains and species (Katoch, 2004). The biggest limitation of the PCR-based approach is the signal to noise ratio in pathological samples, and may lead to a reaction failure if a minimum threshold level of mycobacterial genomic DNA is unavailable in the sample.

Mycobacteriophages, due to their highly specific interaction with mycobacterial host, can be developed as both next-generation therapeutics and diagnostic tools. We and others have previously reported that the cell wall binding domain present in the endolysin protein produced by the phages shows high specificity toward host bacterial cell wall (Kretzer et al., 2007; Schmelcher et al., 2010; Pohane et al., 2014; Yu et al., 2016). Indeed, CBD construct of phage tagged with various reporter proteins such as green, blue, yellow, cyan, and red fluorescent protein have been used to demonstrate its ability to differentiate *Listeria* strains via microscopy (Schmelcher et al., 2010). Similarly unique properties of the CBDs to bind and immobilize *Listeria* cells have been used to recover *Listeria* cells from samples by coating them on paramagnetic beads (Kretzer et al., 2007). In the present study, we have utilized the PG-binding ability of the C-terminal cell wall binding domain of D29 mycobacteriophage LysA to isolate *M. smegmatis cells* from mixed bacterial culture.

We show that the interaction between CTD and *M. smegmatis* cells is highly stable, efficient, and specific. Owing to the presence of outer mycolic acid layer, it was important to carry out Triton X-100 treatment in order to allow CTD to bind directly to *M. smegmatis* cells. We further engineered variants of CTD carrying either GFP or GST that enabled us to exploit its *M. smegmatis* cell wall binding property to develop efficient cell separation and detection assay. For example, our GFP-tagged protein allowed us to visualize *M. smegmatis* directly under a fluorescence microscope. Here, the quantification of GFP fluorescence also suggests that low number of cells can be detected in our assay. We wish to add here that Triton X-100 treatment is essential for the CTD to bind to *M. smegmatis* cells; without Triton X-100 treatment, CTD does not bind *M. smegmatis* (data not shown). To the best of our knowledge, such treatment to directly access the cell wall PG of *M. smegmatis* cell has not been carried out before.

We also generated a GST-tagged CTD protein, which was coated on the Dynabeads by means of anti-GST antibodies. The antibodies against CTD protein were avoided since they will likely interfere in the binding of CTD to the cell wall. Dynabeads can be extracted from the bulk material by magnetic separation. It is this property of Dynabeads that allowed us to capture *M. smegmatis* cells from a suspension containing more than one bacterium. Additionally, CTD-GST-coated Dynabeads can also be stored at 4°C for future use, and are not required to be prepared fresh before every use, thus saving time. *M. smegmatis* after magnetic separation was verified by PCR with primers specific for mycobacterial gene. Very importantly, this method enables us to overcome the major drawback of the PCR-based detection method as discussed above. Thus using CTD, we were able to successfully capture *M. smegmatis* cells from test samples. The designed assay shows 100% sensitivity and specificity toward *M. smegmatis* in comparison with *E. coli* and *B. subtilis*. We have included PCR in this assay as a final confirmation/identification step. Since CTD binds to the *M. tuberculosis* PG also (Pohane et al., 2014), it is worth testing of this designed assay against *M. tuberculosis* and other mycobacterial species to unravel its specificity.

## Data Availability Statement

All datasets generated for this study are included in the article/Supplementary Material.

## Author Contributions

VJ conceived the idea. GN and VJ designed the research, analyzed the data, and wrote the manuscript. GN performed the experiments.

## Conflict of Interest

The authors declare that the research was conducted in the absence of any commercial or financial relationships that could be construed as a potential conflict of interest.

## Supplementary Material

FIGURE S1 SDS-PAGE profiles of the purified recombinant proteins. Panels show the purified CTD, CTD-GFP, and CTD-GST proteins. In each gel, L represents the molecular weight marker with few bands marked.
