# Corrigendum: Separation of *Mycobacterium smegmatis* from a mixed culture using the cell wall binding domain of D29 mycobacteriophage endolysin

**DOI:** 10.3389/fmicb.2022.1033097

**Published:** 2022-09-26

**Authors:** Gokul Nair, Vikas Jain

**Affiliations:** Microbiology and Molecular Biology Laboratory, Department of Biological Sciences, Indian Institute of Science Education and Research, Bhopal, India

**Keywords:** mycobacteria, endolysin, mycobacteriophage, phage therapy, TB diagnostic

Figure 1 in the published article contained an error. The Figure 1 panel A and Figure 2 panel B images have been accidentally duplicated during preparation. More specifically, while this image in Figure 2 is correct, it is incorrect in Figure 1. The corrected Figure 1 and its caption appear below.

**Figure 1:**
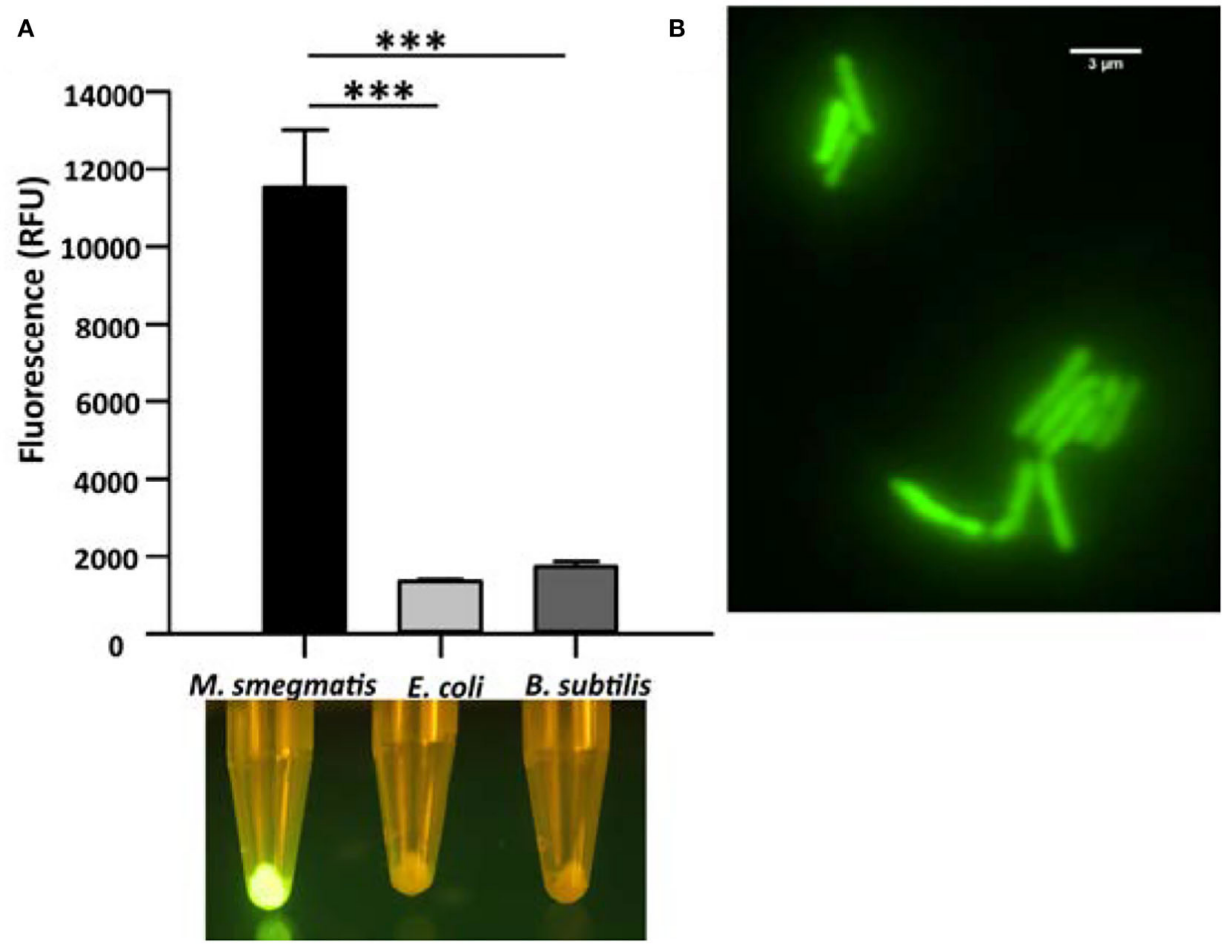
Cell binding assay of CTD-GFP with different bacterial cells. **(A)** The relative fluorescence obtained for CTD-GFP bound to *M. smegmatis, E. coli*, and *B. subtilis* cells. Fluorescence was measured by keeping the excitation and emission wavelengths at 488 and 509 nm, respectively. The data represent an average of three experiments with error bars denoting the standard deviation (*p*-value analysis: ^***^, < 0.0003). The bottom panel shows the image of fluorescing cell pellet obtained after illuminating it with a blue light (~470 nm) source. **(B)** Fluorescence microscopy imaging of CTD-GFP bound *M. smegmatis* cells. The image was taken on a Leica Microsystems fluorescence microscope with a GFP filter.

The authors apologize for this error and state that this does not affect the scientific conclusions of the article in any way. An update has been made to the original article. The original article has been updated.

## Publisher's note

All claims expressed in this article are solely those of the authors and do not necessarily represent those of their affiliated organizations, or those of the publisher, the editors and the reviewers. Any product that may be evaluated in this article, or claim that may be made by its manufacturer, is not guaranteed or endorsed by the publisher.

