# Supplementary figures and images for: Separation of Mycobacterium smegmatis From a Mixed Culture Using the Cell Wall Binding Domain of D29 Mycobacteriophage Endolysin
Source: Front Microbiol. 2020 Jun 5;11:1119. doi: 10.3389/fmicb.2020.01119 (PMC7289928; doi:10.3389/fmicb.2020.01119)

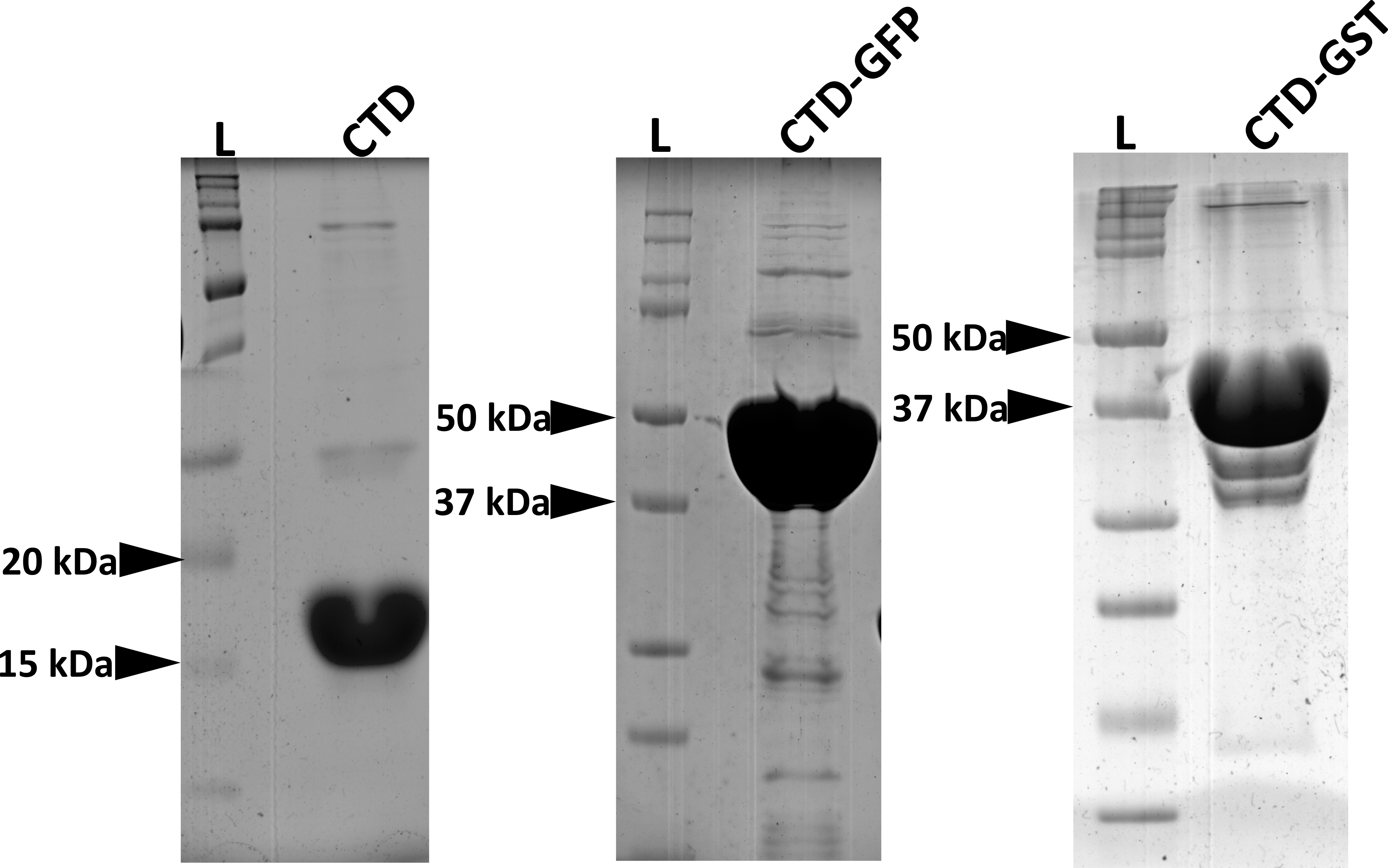

Supplement: FIGURE S1 — SDS-PAGE profiles of the purified recombinant proteins. Panels show the purified CTD, CTD-GFP, and CTD-GST proteins. In each gel, L represents the molecular weight marker with few bands marked. [file Image_1.TIF]
